# Supplementary material for: Towards integration of 64Cu-DOTA-trastuzumab PET-CT and MRI with mathematical modeling to predict response to neoadjuvant therapy in HER2 + breast cancer
Source: Sci Rep. 2020 Nov 25;10:20518. doi: 10.1038/s41598-020-77397-0 (PMC7688955; doi:10.1038/s41598-020-77397-0)
Supplement: Supplementary file 1 — Supplementary Information. [file 41598_2020_77397_MOESM1_ESM.pdf]

Towards integration of ^{64}Cu-DOTA-Trasztusumab PET-CT and MRI with mathematical modeling to predict response to neoadjuvant therapy in HER2+ breast cancer

Angela M. Jarrett[1,2], David A. Hormuth, II[1,2], Vikram Adhikarla[7], Prativa Sahoo[7], Daniel Abler[7], Lusine Tumyan[6], Daniel Schmolze[8], Joanne Mortimer[9*], Russell C. Rockne[7*], Thomas E. Yankeelov[1-5*]

[1]Oden Institute for Computational Engineering and Sciences
[2]Livestrong Cancer Institutes
Departments of [3]Diagnostic Medicine, [4]Oncology, and [5]Biomedical Engineering
The University of Texas at Austin, Austin, Texas, USA

Departments of [6]Radiology, [7]Mathematical Oncology,
[8]Pathology, and [9]Medical Oncology & Therapeutics Research
City of Hope National Medical Center, Duarte, California, USA

*co-senior authors

IHC staining results for each patient where an empirically chosen threshold was applied to the biopsies to identify HER2-overexpressing cancer cells. Note the lack of heterogeneity in HER2 staining of the cells, agreeing with previous studies evaluating the homogeneity of HER2 overexpression in the cancer cells of HER2+ breast tumors [1, 2].

Figure S.1. IHC HER2 expression histology sections for diagnostic biopsies. The first column shows the original slide images for each patient's biopsy. The second column are the same images where all but the biopsy regions have been removed. The third column specifically shows the HER stained cancer cells. As these biopsies were processed in two different facilities, the two image sets show differing staining intensities. However, for both slides HER2-expression is indicated in brown. Note that areas with blue coloring are combinations of adipose and the tumor's supportive stromal tissues. Segmentation of the cancer cells using thresholding indicates that 24% of the total biopsy area is HER2+ for patient 1 and 15% for patient 2. Therefore, for both patients over 10% of the biopsy has intense HER2 staining.

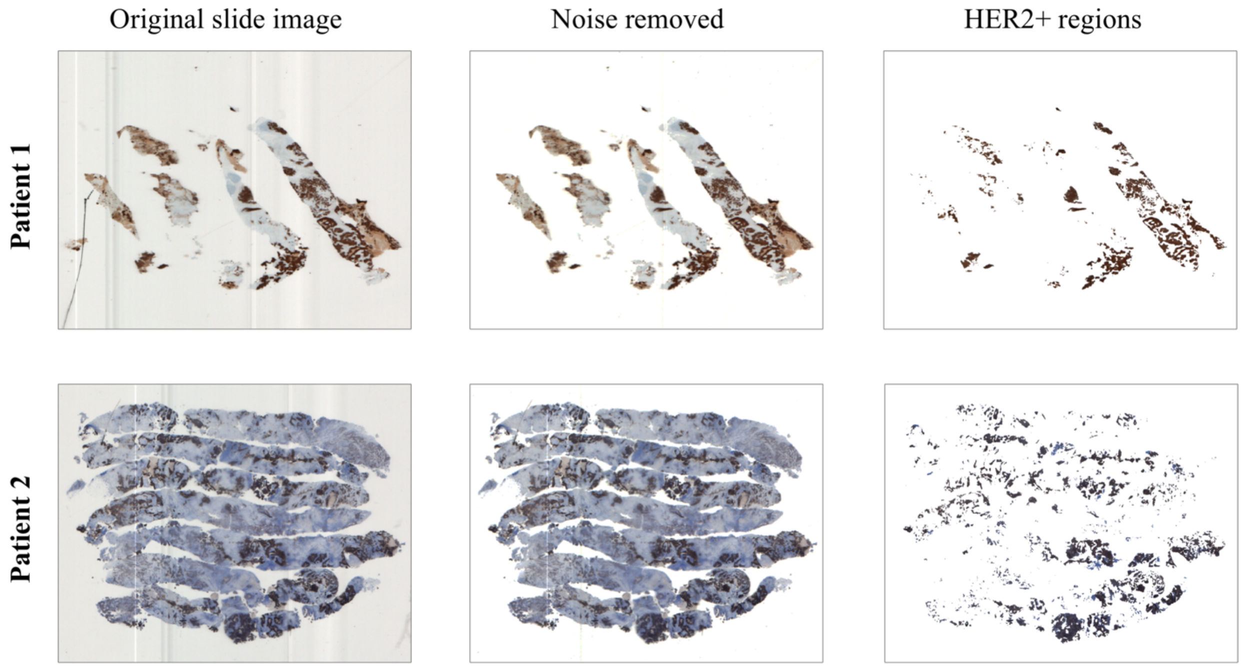

References

1. Press, M.F., et al., *Her-2/neu expression in node-negative breast cancer: direct tissue quantitation by computerized image analysis and association of overexpression with increased risk of recurrent disease.* Cancer Res, 1993. **53**(20): p. 4960-70.
2. Press, M.F., et al., *HER-2 gene amplification, HER-2 and epidermal growth factor receptor mRNA and protein expression, and lapatinib efficacy in women with metastatic breast cancer.* Clin Cancer Res, 2008. **14**(23): p. 7861-70.
